# Supplementary material for: The causal relationship between the human gut microbiota and pyogenic arthritis: a Mendelian randomization study
Source: Front Cell Infect Microbiol. 2024 Nov 26;14:1452480. doi: 10.3389/fcimb.2024.1452480 (PMC11629706; doi:10.3389/fcimb.2024.1452480)
Supplement: Supplementary file 1 [file DataSheet1.zip › mendelian test/ebi-a-GCST90027776.csv_leave_one_out.pdf]

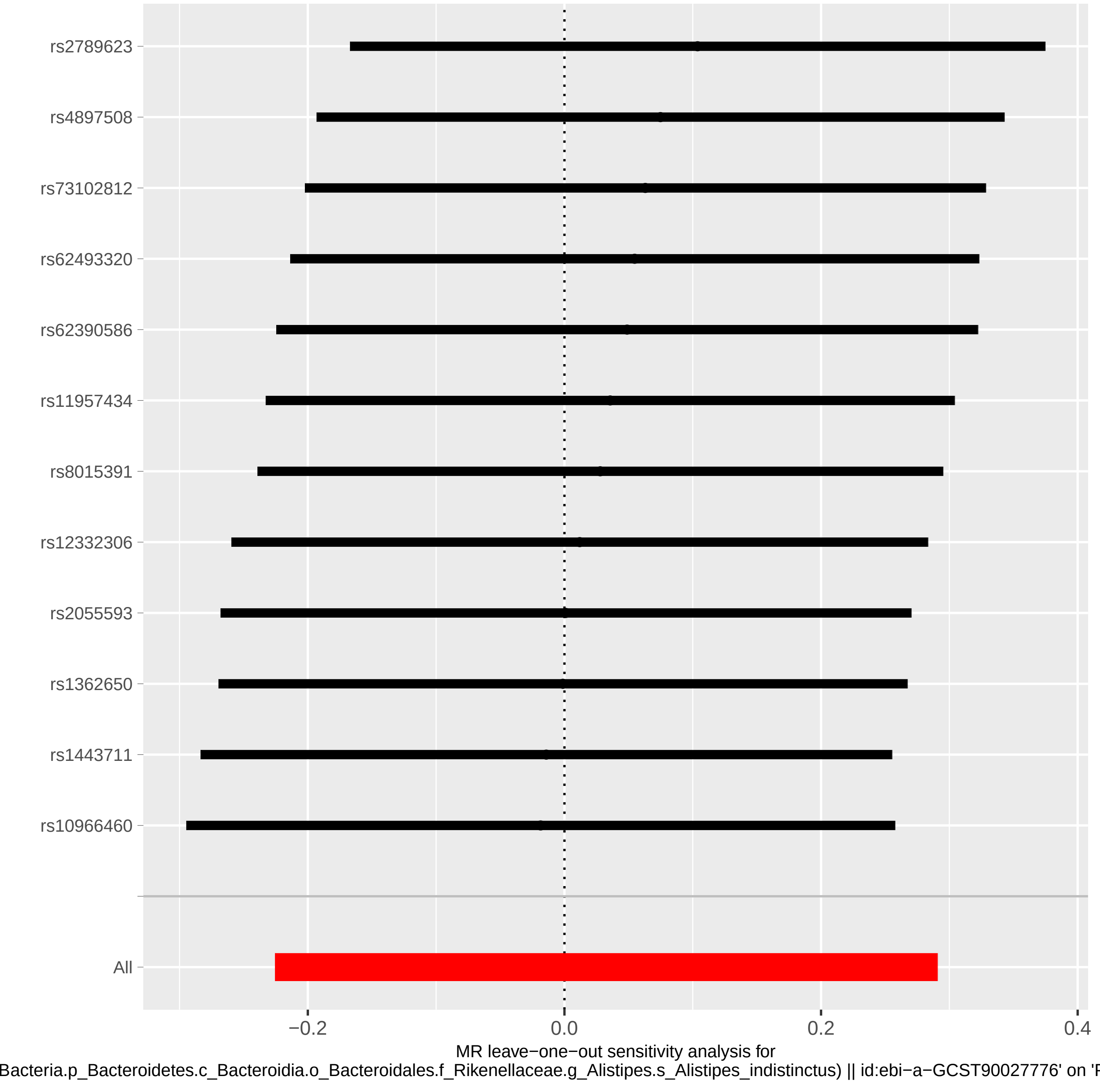
rs2789623
rs4897508
rs73102812
rs62493320
rs62390586
rs11957434
rs8015391
rs12332306
rs2055593
rs1362650
rs1443711
rs10966460
All
−0.2
0.0
0.2
0.4
MR leave−one−out sensitivity analysis for
Bacteria.p_Bacteroidetes.c_Bacteroidia.o_Bacteroidales.f_Rikenellaceae.g_Alistipes.s_Alistipes_indistinctus) || id:ebi−a−GCST90027776' on 'F
